# Color Doppler Score: A New Approach for Monitoring a Large Placental Chorioangioma

**DOI:** 10.1155/2014/723429

**Published:** 2014-09-10

**Authors:** Maria Angelica Zoppi, Alessandra Piras, Ambra Iuculano, Maurizio Arras, Federica Mulas, Maria Carmela Fadda, Sergio Cossu, Giovanni Monni

**Affiliations:** ^1^Department of Obstetrics and Gynecology, Prenatal and Preimplantation Genetic Diagnosis, Fetal Therapy, Microcitemico Hospital, Jenner Street, 09121 Cagliari, Italy; ^2^San Francesco Hospital, Mannironi Street, 08100 Nuoro, Italy

## Abstract

We employed color Doppler score as an innovative approach for the prenatal diagnosis and monitoring of a large placental chorioangioma case diagnosed at 26 weeks and the subjective semiquantitative assessment of the vascularization. The blood flow was assessed by a color Doppler score based on the intensity of the color signal with the following value ranges: (1) no flow, (2) minimal flow, (3) moderate flow, and (4) high vascular flow. Weekly examinations were programmed. Initially, a color Doppler score 3 was assigned, remaining unchanged at the following two exams and decreasing to Score 2 in the following 2 exams and to Score 1 thereafter. The ultrasonographic scan showed an increase of the mass size at the second and third exams and was followed by an arrest of the growth persisting for the rest of the pregnancy. Some hyperechogenic spots inside the mass appeared at the end. Expectant management was opted for, and the delivery was at 39, 2 weeks and maternal and fetal outcomes were favourable. The color Doppler score employed for assessment of vascularization in successive examinations proved to be an important tool for the prediction of the chorioangioma involution, and this new approach of monitoring allowed effective surveillance and successful tailored management.

## 1. Introduction

Large placental chorioangioma are benign proliferations of vessels of the chorionic tissue, with a diameter larger than 4-5 cm. Although very rare (1 : 9,000 to 1 : 50,000 pregnancies), they are frequently associated with fetal and maternal complications often due to possible significant arteriovenous shunts which may lead to polyhydramnios, heart failure, anemia, growth retardation, prematurity, intrauterine fetal death, and mirror syndrome [1–8].

Color Doppler examination can assess the vascular component of placental masses and it is essential for establishing the diagnosis of chorioangioma and differentiating it from other entities such as teratomas or subchorionic hematomas and clots, which are not vascularized [1, 3, 9].

Large chorioangioma may not spontaneously complicate, but, as most complications depend on the entity of vascularization, assessing and monitoring the vascular flow is of value [3, 10]. Close prenatal surveillance and appropriate management may prevent severe complications and perinatal mortality [3, 8, 11].

Semiquantitative assessment of flow by color Doppler imaging is used in gynecological tumors to allow for standardization and score of flow [12, 13]. Subjective assessment is made by using the following score system to describe the amount of flow: Score 1 (no blood flow in the lesion is evident); Score 2 (only a minimal flow can be detected), Score 3 (moderate flow is present), and Score 4 (the mass appears highly vascularized, with marked blood flow). In placental chorioangioma cases, the use of a color Doppler score can facilitate the effective management, by allowing assessment of the magnitude of the flow inside the mass and estimating and monitoring its entity in successive examinations.

We report the use of a color Doppler score as an essential diagnostic tool for semiquantitative assessment and for the monitoring of the vascular component in a large chorioangioma case, in association with the 2D and 3D ultrasound examination. The case was managed expectantly and evolved uneventfully with a favourable neonatal outcome.

## 2. Presentation of the Case

**Figure 1:**
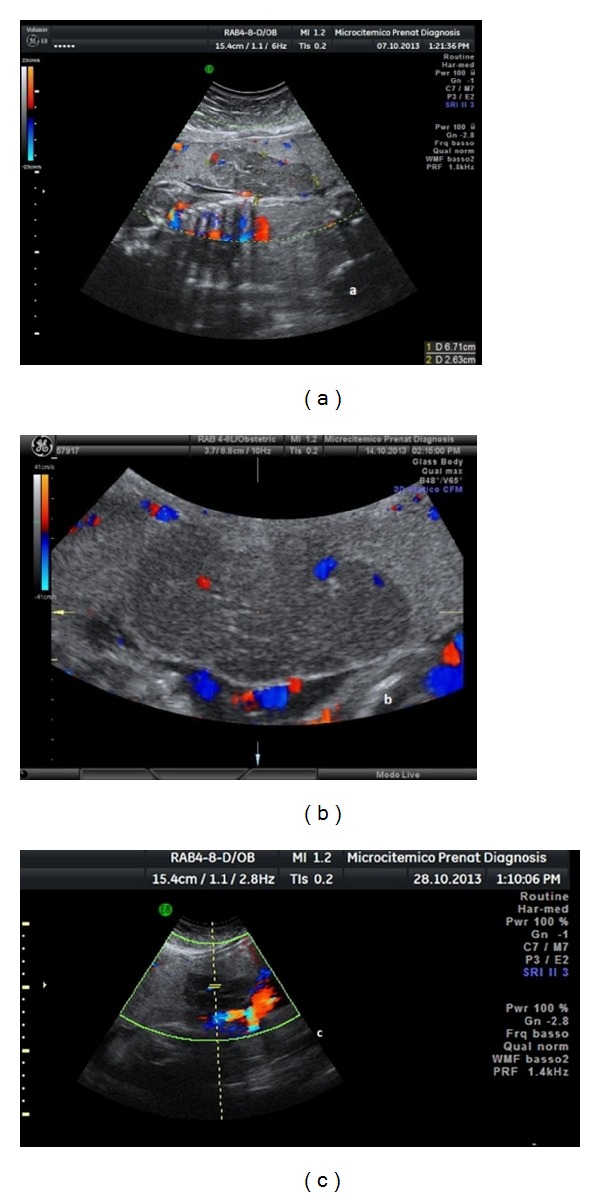
(a) At ultrasound the mass was hypoechogenic, sharply marginated, and circumscribed by a hyperechoic capsule, from which numerous intralesional septa appeared to originate, with some anechoic central areas of vasculature and peripheral vessels (the feeding vessel). Score assigned is 3. (b) Successive examination score assigned is 3. (c) Score assigned is 2.

A 36-year-old primigravida was referred to our centre at 26 + 4 weeks because a placental mass was detected at routine ultrasound examination. The fetal biometry, the umbilical cord, and the amniotic fluid were regular. The hypoechogenic mass was located in the anterior placenta, next to the central umbilical cord insertion, and measured 67 mm × 26 mm × 51 mm. It was marginated and circumscribed by hyperechoic echoes suggestive of a capsule, with numerous septa and some anechoic areas of vasculature (Figure 1). We used 2D and 3D ultrasound, with the latter one giving us the opportunity to measure the axial dimension of the mass.

Color Doppler confirmed that some areas were of low velocity vascularization, synchronous with the fetal heart, and the peripheral feeding vessel. The diagnosis was suggestive of a chorioangioma. We also applied a score based on the intensity of the color Doppler signal within the mass for the subjective assessment of vascularization, as originally proposed for the evaluation of gynecological adnexal masses: Score 1 (no blood flow in the lesion is evident); Score 2 (only a minimal flow can be detected), Score 3 (moderate flow is present), and Score 4 (the mass appears highly vascularized, with marked blood flow) [12, 13]. Assigned color Doppler score was 3 at making the diagnosis. There was no evidence of fetal circulation impairment and it was decided to monitor the pregnancy by weekly exams.

The color Doppler Score remained the same (Score 3) at the following two exams (2nd and 3rd) (Figure 1), then dropped to Score 2 in the following two exams (4th and 5th), and to Score 1 in the successive exams (from the 6th exam on), indicating a progressive reduction of the vascularization.

The ultrasonographic scans showed an increased size of the mass at the 2nd and 3rd exam, followed by an arrest of the growth, which persisted throughout the pregnancy, with the appearance of some hyperechogenic spots inside the mass at the end. The ultrasound devices utilized were GE Voluson E8 and 730 (GE General Electric Healthcare, Italy).

**Figure 2:**
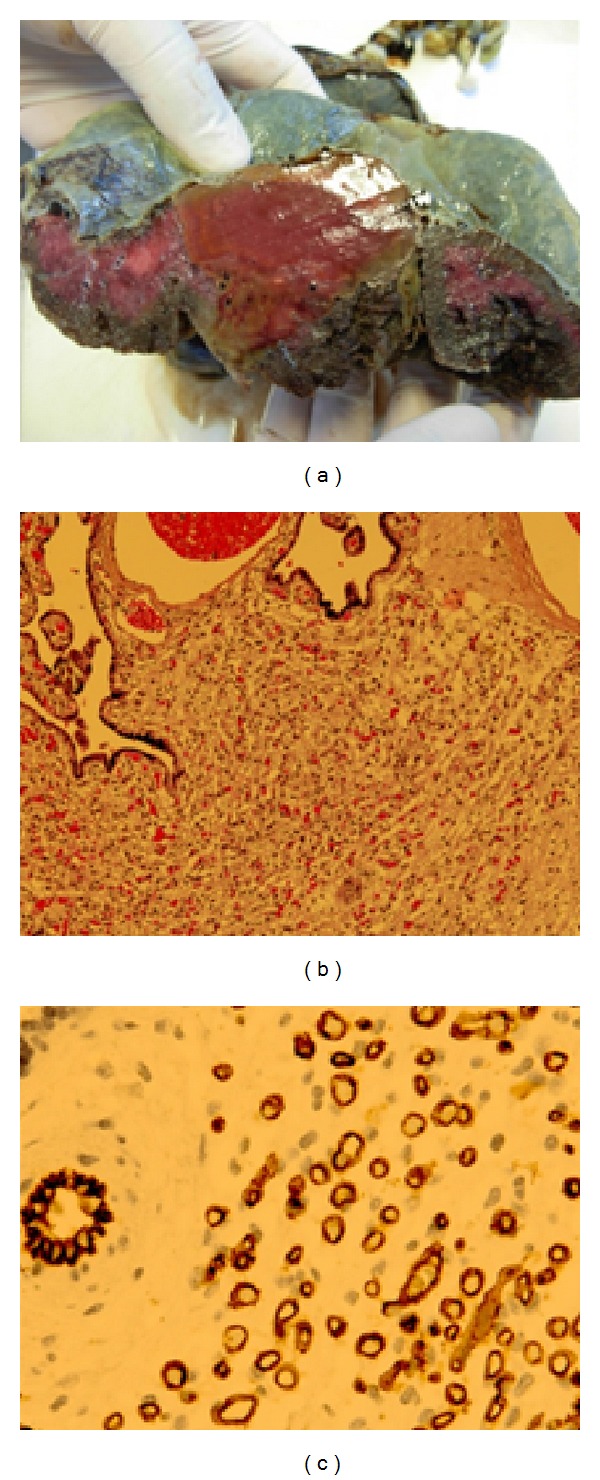
(a) At the cutting the area was of solid appearance with triangular base on the fetal side, near to the cord insertion, with a maximum size of 7 cm. (b) By microscopic examination the lesion was composed of proliferation of capillary structures of small caliber, single coated with endothelial cells (100x). (c) The CD31, a specific marker of endothelial cells, puts greater emphasis on the typical structure of a placental chorioangioma (400x).

The patient had a vaginal delivery by vacuum extractor at 39 + 2 weeks, giving birth to a female newborn weighing 3,460, grams without any perinatal complications. At the macroscopic examination of the placenta (Figure 2) the mass measured 7 cm and was situated close to the three-vessel umbilical cord. Microscopically, the lesion was composed of a proliferation of small-caliber capillary structures, single coated with endothelial cells (Figure 2) and with disseminated intraluminal thrombosis, ischemic necrosis, and microcalcifications, without a detectable capsule but surrounded by a stromal myxoid component. Positivity for CD31 and CD34 endothelial cells markers was detected as well as focal positivity for the cytokeratin 18 marker, leading to the diagnosis of placental hemangioma (chorioangioma).

## 3. Discussion

In our case of large chorioangioma the color Doppler score was used for the assessment of vascularization in successive examinations, proved to be a valuable tool for the prediction of involution of the chorioangioma, and allowed for effective surveillance and tailored management.

The color Doppler was useful for establishing the diagnosis and for differentiating diagnosis from other placental masses such as hematoma, partial hydatiform mole, and teratoma [1, 3, 9]. This efficient approach aided us in the subjective assessment of the vascularization based on the score on the intensity of the color Doppler signal within the mass and allowed us to detect a reduction in the vascularity parallel to the involution of the mass.

Despite the large size of the chorioangioma and the presence of vascularization at the initial observation, the subsequent monitoring showed a reduction of the vascularization and provided good prognosis signs, leading to the applying more confident expectant management.

Not finding other similar case reports in the existent literature, our team is the first one to employ the method of subjective semiquantitative assessment of vascularization by a color Doppler score for chorioangioma monitoring, which has generally been adopted so far for monitoring gynecologic adnexal masses only [12, 13].

The 3D ultrasound scan helped us measure the axial dimension of the mass. Our ultrasound exams suggested the presence of a true capsule around the mass, which had however the microscopic correspondence of a stromal myxoid accumulation.

Prenatal treatment of the chorioangioma is still controversial. Expectant management by serial ultrasound is the treatment choice for small and asymptomatic tumors and fo large tumors without clinical complication. If complications develop in late pregnancy and the fetus has reached a good maturity, planned delivery could be considered. If polyhydramnios occurs, amnioreduction or transplacental therapy with COX-2 inhibitor must be taken into account as well as intrauterine fetal blood transfusion for the treatment for fetal anemia [3–11]. Antenatal embolization of the feeding vessel or of the vascular shunts by endoscopic laser coagulation, alcohol sclerosant injection, or microcoil embolization has been proposed. If the fetus develops signs of cardiac failure, cesarean section can be adopted to reduce neonatal complications [11].

A systematic review was performed recently comparing antenatal complications and outcomes between no prenatal treatment and in utero treatment including 64 articles reporting 112 cases of chorioangioma; it could suggest that overall treatment and antenatal intervention may improve the outcome, but due to the small number of cases, no conclusion was made about the benefit of treatment over conservative management [5].

Most of the complications of chorioangioma have a circulatory origin, linked to a fetal cardiac overload. The evaluation with the color Doppler score enabled us to be more confident in the assessment of the vascular component, which preceded the total volume involution of the mass and the deposition of calcium, rendering the monitoring easier and safer. We think that the use of this simple management will assist clinicians in the monitoring of chorioangioma cases, even those who develop complications.

In this case of large chorioangioma, the color Doppler score used for assessment of vascularization proved to be an important tool for the prediction of involution of the chorioangioma, allowing for effective surveillance and successful tailored management.
